# iPBAvizu: a PyMOL plugin for an efficient 3D protein structure superimposition approach

**DOI:** 10.1186/s13029-019-0075-3

**Published:** 2019-11-02

**Authors:** Guilhem Faure, Agnel Praveen Joseph, Pierrick Craveur, Tarun J. Narwani, Narayanaswamy Srinivasan, Jean-Christophe Gelly, Joseph Rebehmed, Alexandre G. de Brevern

**Affiliations:** 1 INSERM, U 1134, DSIMB, Univ Paris, Univ de la Réunion, Univ des Antilles, F-75739 Paris, France; 2 INSERM UMR_S 1134, DSIMB, Université de Paris, Institut National de la Transfusion Sanguine (INTS), 6, rue Alexandre Cabanel, F-75739, Paris cedex 15, France; 3 Laboratoire d’Excellence GR-Ex, F-75739 Paris, France; 4 Birkbeck College, University of London, London, UK; 5 Molecular Graphics Laboratory, Department of Integrative Structural and Computational Biology, The Scripps Research Institute, La Jolla, CA 92037 USA; 6 Molecular Biophysics Unit, Indian Institute of Science, Bangalore, India; 7 Department of Computer Science and Mathematics, Lebanese American University, Beirut, Lebanon

**Keywords:** Protein superimposition, Structural alphabet, Visualisation, Structural alignment, Structural bioinformatics

## Abstract

**Background:**

Protein 3D structure is the support of its function. Comparison of 3D protein structures provides insight on their evolution and their functional specificities and can be done efficiently via protein structure superimposition analysis. Multiple approaches have been developed to perform such task and are often based on structural superimposition deduced from sequence alignment, which does not take into account structural features. Our methodology is based on the use of a Structural Alphabet (SA), i.e. a library of 3D local protein prototypes able to approximate protein backbone. The interest of a SA is to translate into 1D sequences into the 3D structures.

**Results:**

We used Protein blocks (PB), a widely used SA consisting of 16 prototypes, each representing a conformation of the pentapeptide skeleton defined in terms of dihedral angles. Proteins are described using PB from which we have previously developed a sequence alignment procedure based on dynamic programming with a dedicated PB Substitution Matrix. We improved the procedure with a specific two-step search: (i) very similar regions are selected using very high weights and aligned, and (ii) the alignment is completed (if possible) with less stringent parameters. Our approach, iPBA, has shown to perform better than other available tools in benchmark tests. To facilitate the usage of iPBA, we designed and implemented iPBAvizu, a plugin for PyMOL that allows users to run iPBA in an easy way and analyse protein superimpositions.

**Conclusions:**

iPBAvizu is an implementation of iPBA within the well-known and widely used PyMOL software. iPBAvizu enables to generate iPBA alignments, create and interactively explore structural superimposition, and assess the quality of the protein alignments.

## Background

The detection of structural analogy between protein folds requires development of methods and tools to compare and classify them. This is extremely helpful for studying evolutionary relationships between proteins especially in the low sequence identity ranges [1]. However, an optimal superposition is far from being a trivial task. Popular methods such as DALI [2] and CE [3], use a reduced representation of backbone conformation in terms of distance matrices.

Protein backbone conformation can be characterized by a set of local structure prototypes, namely Structural Alphabets (SAs), which enables the transformation of 3D information into a 1D sequence of alphabets [4]. Hence a 3D structure comparison can be obtained by aligning sequences of SAs (protein structures encoded in terms of SA). A SA consisting of 16 pentapeptide conformations, called Protein Blocks (PBs), was developed in our group [5]. Based on this library, a protein superimposition approach was developed. A substitution matrix for PBs [6] was generated based on all PB substitutions observed in pairwise structure alignments in PALI dataset [7]. The superimposition was carried out with simple dynamic programming approaches [8]. We recently improved the efficiency of our structural alignment algorithm by (i) refining the substitution matrix and (ii) designing an improved dynamic programming algorithm based on preference for well-aligned regions as anchors. This improvement (improved Protein Block Alignment, iPBA) resulted in a better performance over other established methods like MUSTANG [9] for 89% of the alignments and DALI for 79% [10]. Benchmarks on difficult cases of alignment also show similar results [11, 12]. Protein Blocks were also recently used to analyse Molecular Dynamic simulations [13, 14] underlining their abilities to apprehend protein flexibility [15].

We present here a plugin, iPBAvizu, which integrates the efficient protein structure alignment approach iPBA with the very popular molecular graphics viewer PyMOL (The PyMOL Molecular Graphics System, Version 1.7, Schrödinger, LLC) from which several plugins like PyKnoT [16] or PyETV [17] have been integrated in. iPBAvizu enables interactive visualization and analysis of protein structure superposition and the resulting sequence alignment. Different scores to assess the quality of the alignment are also given.

## Results

After installing all the dependencies, iPBAvizu can be easily integrated within PyMOL using the ‘Plugin’ menu on the PyMOL console, choosing ‘Install’ under the ‘Manage Plugins’ and then locating and selecting the iPBAvizu.py file. The installation procedures as well as few examples of structural alignments are illustrated in a series of videos (see http://www.dsimb.inserm.fr/dsimb_tools/iPBAVizu/). The plugin is easy to use and does not require any command line or programming skills. It is fully controlled by the PyMOL GUI.

To launch iPBAvizu from the PyMOL Wizard menu, at least two protein structures must be loaded and made available in the PyMOL session. iPBAvizu menu appears in PyMOL GUI, like the Measurement or Fit native functions. Users can select two chains among the available loaded structures, and then select ‘Align!’ to run iPBA program. Once the alignment process is over, results are displayed as two new protein objects in PyMOL. The two new objects correspond to the two aligned structures. A new window containing different alignment scores (e.g., GDT-TS, RMSD, see Methods) and an interactive sequence alignment manager is also displayed. Both residue and Protein Block sequences of aligned structures are given. Users can highlight any residue or PB of one or both sequences. Highlighting selects the residues directly in the 2 new aligned protein objects created in PyMOL 3D window. This interactive functionality provides an efficient way to explore sequence and structural alignment.

**Fig. 1:**
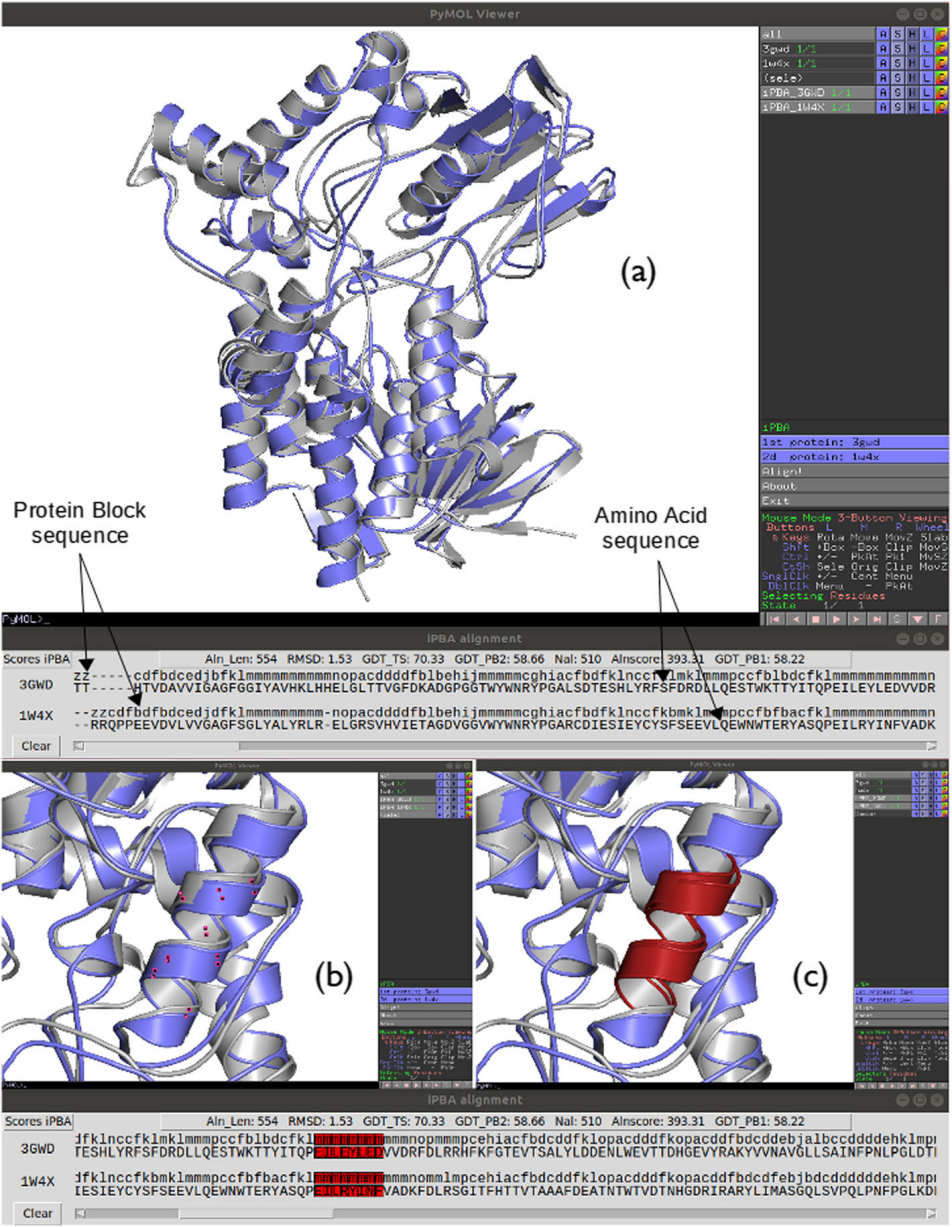
*Example of iPBAvizu usage*. (**a**) Two proteins, with lengths of 531 and 533 residues respectively are loaded into PyMOL (PDB code: 3GWD and 1W4X respectively); the structural superimposition is made using iPBAvizu. Arrows show the position of Amino acid and Protein Block sequence. This independent window contains the sequence alignment in terms of residues and PBs with different scores. It allows an interactive selection between the sequences and the structures. In the right panel are shown the two loaded proteins, then the two superimposed chains (the prefix iPBA_ is added before their names) and finally a select case, this last is not necessary but for some PyMOL versions must be shown (please do not interact with it without necessity). (**b**) and (**c**) show the selection of a protein fragment and rendering when a specific color is chosen

Figure 1 shows an example of structural superposition of two proteins of the monooxygenase protein family using iPBAvizu plugin: Cyclohexanone Monooxygenase (CHMO, PDB code 3GWD) and Phenylacetone Monooxygenase (PAMO, PDB code 1W4X) [18]. The obtained results were also compared with other popular superimposition tools (e.g., cealign [3] and TM-align [19]). The alignment generated by iPBA based on PBs was compared to alignment generated with cealign and TM-align and the iPBA alignment show a better Cα RMSD score (1.5 Å versus values between 1.9–2.7 Å for the 2 other approaches). The values are provided for the aligned residues that are on average larger than with other superimposition tools.

## Discussion & Conclusion

A structural alphabet is a library of protein fragments able to approximate every part of protein structures (for a review [20]). These libraries yielded prototypes that are representative of local folds found in proteins. The structural alphabet allows the translation of three-dimensional protein structures into a series of letters. As a result, it is possible to use classical sequence alignment methodologies to perform structural alignments. The main difficulty lies in obtaining a pertinent substitution matrix that gives the similarity score between alphabets, which guides the alignments. Few teams have used this approach to perform structural comparisons and/or PDB mining:

Guyon and co-workers had used a structural alphabet based on Hidden Markov Model and proposed an approach named SA-search (http://bioserv.rpbs.jussieu.fr/cgi-bin/SA-Search, [21]). Their substitution matrix is generated from a transition matrix, however the details of the method are uncleared. The webserver gives only C-alpha coordinates for superimposition and does not provide a fully interactive interface to explore structural alignment. Finally, SA-Search webserver has not been updated since 2006 and miss modern web-technology interactivity based.

3D-BLAST was developed late 2006 and is based on the BLAST methods [22]. The structural alphabet proposed is based on optimization of nearest-neighbor clustering (NNC). Interestingly the substitution matrix was generated based on SCOP classification. Since 3D-BLAST was initially developed to search for structural similarity and not to specifically compare two protein structures of interest, it was not benchmark. The webserver (http://3d-blast.life.nctu.edu.tw/) needs Chime applet, and users do not have a direct access to simple alignment results.

SA-FAST was developed for the same purpose [23] but was based on FASTA algorithm. Structural alphabet was generated using a Self-Organizing Map, taking into account the most frequent clusters. The final benchmark was done using 50 proteins. The webserver (http://bioinfo.cis.nctu.edu.tw/safast/) is very fast. However, it is not possible to do simple pairwise alignments and the output needs Chime applet which is not very easy to install. The major drawback is that users do not have access to the alignment by itself for further analysis.

CLePAPS [24] is based on the use of a dedicated structural alphabet built only to perform database search. In the first step, aligned fragment pairs (AFP) are found, which correspond to fragments that involve exact matches of similar letters. CLePAPS then joins consistent AFPs guided by their similarity scores to extend the alignment by several “zoom-in” iteration steps; it does not use dynamic programming. CLePAPS was tested on a limited number of protein structure pairs. A stand-alone program is reported to be available, but not found.

Hence, iPBAvizu is quite interesting approach. Indeed, it is an easy-to-use plugin for PyMOL that allows users to superimpose protein structures using iPBA methodology, an efficient way to superimpose protein 3D structures [11] and explore the structural alignment results. Its total integration as a plugin into PyMOL molecular viewer offers an easy but powerful way to process and study structural alignment with quantitative measurements.

## Materials and methods

iPBA program is fully written in Python (2.7+). It depends on ProFit program stand-alone version (Martin, A.C.R., http://www.bioinf.org.uk/software/profit) for generating the final structural alignment. iPBA provides an efficient way to align two protein structures using anchor-based alignment methodology [11, 12].

iPBAvizu package has an installer to configure iPBA and manage its dependencies on the local machine before integrating it into PyMOL. Due to ProFit requirements, iPBAvizu is only available on Unix-based operating systems. iPBAvizu is embedded into PyMOL as a wizard plugin, and all iPBA functionalities are totally integrated into the graphic interface of PyMOL. iPBAvizu can be launched with the current PyMOL internal GUI. Users can easily align structures with a few clicks and access both scores and the alignment results that are displayed in PyMOL itself, as a Tkinter GUI. The alignment window is interactive; it is linked to 3D PyMOL interface for the best interpretation and exploration of results.

iPBA and iPBAvizu can estimate the quality of the superimposition via a score. The GDT score (GDT_TS) is widely used for the assessment of structural models generated in CASP structure prediction trials [25], it is supposed to be less sensible to large deviation as seen with Root Mean Square Deviation (RMSD). The GDT_TS is the combination of set of superimposed residues for fixed thresholds at 1, 2, 4 and 8 Å. GDT_PB scores (calculated in a similar way as that of GDT_TS, but using PB substitution scores [11, 12] instead of distances) are also provided for the hits obtained (see for [11, 12] more details).

Protein Blocks (PB) and amino acid sequences are provided. PB is the most widely used structural alphabet and is composed of 16 local prototypes [4] of five residue length, it is dedicated to analyse local conformations of protein structures from the Protein DataBank (PDB) [26]. Each PB is characterized by the φ and ψ dihedral angles of five consecutive residues. PBs give a reasonable approximation of all local protein 3D structures [14, 27, 28]. PBs are labelled from *a* to *p*. PBs *m* and *d* can be roughly described as prototypes for α-helix and central β-strand, respectively. PBs *a* to *c* primarily represent β-strand N-caps and PBs *e* and *f* representing β-strand C-caps; PBs *g* to *j* are specific to coils; PBs *k* and *l* to α-helix N-caps while PBs *n* to *p* to α-helix C-caps. For each PB is associated 5 residues, its assignment is done on the central residue. As PBs are overlapping, a structure of length *N* is translated in *N*-4 PBs, the two first and two last residues are associated to letter Z (see Fig. 1). Missing residues are also associated to the letter Z.

## Data Availability

iPBAvizu is a PyMOL plugin freely available to the academic scientific community, i.e. the data is only informatics codes. It is composed of the PyMOL script code and the iPBA code. This last used python and some C codes. The downloadable archive can be freely accessed at our academic website: http://www.dsimb.inserm.fr/dsimb_tools/iPBAVizu/. As it is a PyMOL plugin, user needs to install independently PyMOL software: https://pymol.org. There is no restriction for use or modifications of iPBAvizu by any academic scientists. For commercial usage, please contact the authors.
